# Framework for fusing traffic information from social and physical transportation data

**DOI:** 10.1371/journal.pone.0201531

**Published:** 2018-08-02

**Authors:** Zhihao Zheng, Chengcheng Wang, Pu Wang, Yusha Xiong, Fan Zhang, Yisheng Lv

**Affiliations:** 1 School of Traffic and Transportation Engineering, Central South University, Changsha, Hunan, China; 2 Shenzhen Institutes of Advanced Technology, Chinese Academy of Sciences, Shenzhen, Guangdong, China; 3 The State Key Laboratory of Intelligent Control and Management of Complex Systems, Institute of Automation, Chinese Academy of Sciences, Beijing, China; Beihang University, CHINA

## Abstract

Tremendous volumes of messages on social media platforms provide supplementary traffic information and encapsulate crowd wisdom for solving transportation problems. However, social media messages manifested in human languages are usually characterized with redundant, fuzzy and subjective features. Here, we develop a data fusion framework to identify social media messages reporting non-recurring traffic events by connecting the traffic events with traffic states inferred from taxi global positioning system (GPS) data. Temporal-spatial information of traffic anomalies caused by the traffic events are then retrieved from anomalous traffic states. The proposed framework successfully identified accidental traffic events with various scales and exhibited strong performance in event descriptions. Even though social media messages are generally posted after the occurrence of anomalous traffic states, resourceful event descriptions in the messages are helpful in explaining traffic anomalies and for deploying suitable countermeasures.

## Introduction

Recent rapid developments in sensing and communicating techniques have facilitated the boom of big transportation data [1,2]. Many novel analytic and modeling approaches have been proposed in this context [3,4]. The transportation data used in existing research and practices were usually collected using sensing devices installed on vehicles or roads. Typical examples include real-time coordinates of taxies and buses collected by onboard global positioning system (GPS) signal receivers [5–7], traffic speed information from remote microwave sensor [8] and the traffic count information collected by loop detectors [9–11] or video cameras [12–14]. Big transportation data have been widely applied in the estimation of travel demand [15–17], transit passenger flow [18,19] and the management of transportation systems [20–22].

Given that traffic information in most big transportation data is collected using the physically installed sensing devices, here we call them physical transportation data according to the manner of data collection. Despite the good data quality and wide availability of physical transportation data, one common disadvantage of such data is the lack of information on the understanding and analysis of traffic states. For example, taxi GPS data are widely used in estimating vehicle speeds on roads. However, when the abnormally slow vehicle speed is detected, the GPS data themselves cannot show whether the slow vehicle speed is caused by traffic congestion or a traffic accident. Supplementary information is needed to better understand and interpret the sensed traffic states, and to design a more suitable management strategy.

There are many social media platforms, such as Twitter in the U.S. and Weibo in China [23]. A significant number of social media users frequently post and spread information on every aspect of their lives, among which transportation is a frequently discussed topic. These kinds of transportation data are generated by social media users in a crowdsourcing manner, here we call them social transportation data. Social transportation data can offer supplementary traffic information for physical transportation data. For example, social media messages usually incorporate descriptions of traffic incidents, and the messages may record the possible reason of a traffic incident or include suggestions on transportation infrastructure planning and transportation management.

Based on social transportation data, methods for mining traffic information and detecting traffic anomalies have been investigated in recent years. Wang *et al*. [24,25] proposed that social signals comprise one of the most important data sources for parallel transportation and control of complex transportation systems. Ni *et al*. [26] developed a systematic approach to examine the correlation between social media activities and social event occurrences. The authors found a positive correlation between passenger flow and the rates of social media posts. Mo *et al*. [27] used the theories of linguistic dynamic systems to analyze traffic information from traffic police micro-blogs. Zeng *et al*. [28] employed social network analysis methods to study the topic evolution pattern on social media platforms. The authors found that social media are more applicable in sensing road emergencies than traditional media. Chen *et al*. [29] claimed that social media tweets can act as sensors to monitor traffic congestion. Kuflik *et al*. [30] proposed a framework for mining transport-related information from social media data, and discussed the potential and the challenges in social media data processing.

Motivated by such encouraging discoveries, several researchers have mined various types of traffic information from social transportation data. Some researchers have focused on a systematic approach for processing social media data, while others have focused on specific problems, such as classifying transport-related information, recognizing location entities, etc. Georgakis *et al*. [31] developed a framework for real-time detection of traffic events including the classifying and geolocating processes. Wang *et al*. [32] proposed a traffic alert and warning system using the latent Dirichlet allocation method to identify transport-related social media data. Gu *et al*. [33] mined social media tweets to extract traffic incident on both highways and arterials by mapping tweets into a high-dimensional binary vector. Zhang [34] proposed a hybrid mechanism to model incident-level semantic information from social media data. Fu *et al*. [35] collected tweets with real-time traffic information, and filtered redundant tweets to generate concise and comprehensible textual contents. D’Andrea *et al*. [36] identified transport-related tweets from Twitter streams using a support vector machine (SVM) algorithm, and developed a real-time traffic monitoring system to detect traffic events. Tejaswin *et al*. [37] proposed a novel location entity extraction method using background knowledge, and built an automated social media analysis system to assist traffic management. Most recently, a deep learning approach proposed by Zhang *et al*. [38] was used to detect traffic accidents in social media data. The authors compared the results with accident log and loop detector data, finding that accident-related tweets can be located by most accident log and nearby traffic anomalies.

Learning human mobility from social media data is also a hot topic in this field of research. Trip purpose, mode of transport, activity duration, and destination choice, as well as land uses, have been widely studied [39]. Jiao *et al*. [40] discovered the relationship between geo-tagged tweets and a special event, finding that social media can serve as a monitoring tool to learn future special events. Zhang *et al*. [41] proposed a sequential-model-based clustering method to infer the longitudinal travel behavior from geo-tagged social media data. Using similar data, Hu *et al*. [42] presented a dynamic model to estimate time-of-day trip arrival patterns at a fine-grained level, and Hasan *et al*. [43] inferred individual activity patterns from large-scale geo-located social media data using a topic modeling classification method. Based on user-generated data from social media, Gkiotsalitis *et al*. captured users’ willingness to travel a certain distance to participate in different types of activities using a utility maximization model [44], and derived an individual’s preferences on the location of a joint leisure activity and arrival times from a perceived utility model and a stochastic annealing search method [45].

To visualize traffic information in social transportation data, several information visualization platforms were developed; examples include the classification analysis systems and Android-based apps [46–48]. Cui *et al*. [49] collected traffic information from social media interactions and developed an Android-based app to capture and broadcast traffic status information. Lécué *et al*. [50–52] developed a system named STAR-CITY to analyze, diagnose, explore, and predict traffic states using semantic web technologies. Recently, Zheng *et al*. [1] reviewed the data sources, analytical approaches, and application systems of social transportation research, and stated that social transportation may be the basis of next-generation intelligent transportation systems.

Most previous research has focused on developing advanced semantic analysis tools and automated systems to retrieve more information from social media to monitor traffic states. However, the real value of social transportation data is to provide reasoning descriptions rather than taking full responsibility for traffic monitoring. We should focus more on discovering valuable social transportation data and obtain more in-depth traffic information by connecting them with physical transportation data. So far, in only a few works have researchers tried to discover the potential of combining two types of data sources. Pan *et al*. [53] presented a method to detect traffic anomalies according to routing behavior derived from GPS data, and provided descriptions for anomalies by mining terms from social media data afterwards. Wang *et al*. [54] proposed a coupled matrix and tensor factorization model to fuse traffic information from GPS probe data and social media data. These pioneering studies proved the vast potential of incorporating information from social transportation data and physical transportation data.

In this study, we propose a data-fusion framework to detect and describe non-recurring traffic anomalies reported by social media data. Here, social media data contribute as an initial filtering of potentially critical traffic events since such events are more likely to be reported by people. Traffic states from taxi GPS data are used to identify non-recurring traffic events and provide temporal-spatial information of caused anomalies. We can also describe the causes of the anomalies with social media data. To identify non-recurring traffic events, we propose an anomaly detection method using historical GPS data to find anomalous travel paths in generated searching regions. This framework does not require an exact location from social media data but utilizes accurate taxi GPS data to pinpoint the time and location of the traffic anomaly, which minimizes the possibility of missing important social transportation messages. We suppose that the present work can release more potential of social transportation data, consequently promoting the development of social transportation studies and extending the application of social transportation data in practices.

## Data

Here, we describe the social transportation data and physical transportation data used in this study. The physical transportation data were generated by the GPS receivers of taxies during workdays in October 2014 (19 days in total). The social transportation data are generated by social media users of Sina Weibo during the same observation period. All taxi probe readings were provided by a third-party organization and all car plates were anonymized by unique identifiers. All Sina Weibo data were collected according to Sina Weibo’s terms of service and privacy conditions.

### Social transportation data

The social transportation data were collected from the largest micro-blog platform in China, Sina Weibo. The social media platform, Weibo, has 230 million active users, which represents one-sixth of the Chinese population. Weibo messages usually record users’ descriptions, complaints, and suggestions regarding traffic and transportation. We used the transport-related keywords shown in Table 1 to collect transport-related messages from Weibo. A web crawler was developed to automatically collect Weibo messages that contain these keywords (Table 2). The post-time information, the message, and the location tag that was voluntarily uploaded by users were recorded. However, a location tag is not accompanied by a geographical coordinate, and only a small proportion of Weibo posts are geo-tagged. Weibo messages containing transport-related keywords may not actually reflect traffic events. We manually identified 601 transport-related Weibo messages from all Weibo messages collected. The two types of Weibo messages are shown in Table 2.

**Table 1:** Keywords list.

Traffic	Accident	Car	Vehicle
Road	Congestion	Street	Avenue

**Table 2:** Examples of two types of Weibo data in standard format.

Post time	Text (translated into English)	Location tag	Type
11:28 Oct. 17	At 11:25, an accident occurred at South Futian Road and Binhe Avenue, occupied the middle lane, and caused congestion.	Not applicable	Road name
8:40 Oct. 8	At 8:30, a car crash accident at Buji entrance slowed traffic.	Buji entrance	Landmark

### GPS data

We used taxi GPS data collected by 13,584 taxies equipped with GPS receivers. In the observation period (19 workdays in October 2014), a total of 916,851,503 GPS readings were recorded. On average, a GPS record is collected every 15 s for each taxi. GPS data are discarded in the following cases: (1) GPS records collected when taxi drivers search passengers. In this case, taxi drivers intend to drive slower. (2) GPS records of taxies with more than 500 passenger trips recorded during 1 d, which is usually caused by device failures. (3) GPS records collected beyond urban active hours (6:00–23:00). Finally, 255,490,492 taxi GPS readings were selected and used in this study. High-resolution road network data provided by the Shenzhen Transportation Authority were also employed in this study. The road network is composed of 85,389 nodes (breakpoints to determine the geographical shapes of the roads) and 116,642 road segments. The road network data also incorporate attributes of road segments, which include length, speed limit, number of lanes, and road class.

## Method

### Map matching of GPS data

We used the ST-matching algorithm [55] to map GPS probe readings to road segments. In the following, we describe the method in detail.

(1) Determining candidate points: For a trajectory point *p*_*i*_ in a GPS trajectory *TR* = *p*_1_ → *p*_2_ → ⋯ → *p*_*n*_, we select candidate road segments with perpendicular distances *d* ≤ 35 *m* to *p*_*i*_ (the widest road (one direction) in Shenzhen is 32 m). The candidate point $c_{i}^{j}$ of the trajectory point *p*_*i*_ is defined as the intersection of candidate road segment *j* with the perpendicular line from *p*_*i*_ to *j*. Trajectory points without candidate segments are eliminated from the GPS trajectory *TR*.

(2) Spatial analysis function: Observation probability $f(c_{i}^{j})$ is the likelihood of matching the trajectory point *p*_*i*_ to the candidate point $c_{i}^{j}$ based on the distance $x_{i}^{j}=dist(c_{i}^{j},p_{i})$:

$$f(c_{i}^{j})=\frac{1}{\sqrt{2\pi }\sigma }e^{-\frac{{\left(x_{i}^{j}-\mu \right)}^{2}}{2\sigma^{2}}}(\mu =0,\sigma =20\;m)$$ (1)

where *σ* = 20 m is the standard spatial deviation of GPS readings. Transmission probability $P(c_{i-1}^{t}\to c_{i}^{s})$ is the likelihood of considering the path between two neighboring candidate points $\left(c_{i-1}^{t},c_{i}^{s}\right)$ as the shortest path:

$$P(c_{i-1}^{t}\to c_{i}^{s})=\frac{d_{i-1\to i}}{w_{\left(i-1,t)\to (i,s\right)}}$$ (2)

where *d*_*i*−1→*i*_ = dist(*p*_*i*_,*p*_*i*−1_) is the Euclidean distance between trajectory point *p*_*i*−1_ and trajectory point *p*_*i*_, and *w*_(*i*−1,*t*)→(*i*,*s*)_ is the length of the shortest path from candidate point $c_{i-1}^{t}$ to candidate point $c_{i}^{s}$. Integrating transmission probability and observation probability, the spatial analysis function $F(c_{i-1}^{t}\to c_{i}^{s})$ is defined as:

$$F(c_{i-1}^{t}\to c_{i}^{s})=P(c_{i-1}^{t}\to c_{i}^{s})\times f(c_{i}^{j}),\;2\leq i\leq n$$ (3)

We then calculate $F(c_{i-1}^{t}\to c_{i}^{s})$ for all candidate paths between neighboring trajectory points *p*_*i*−1_ and *p*_*i*_.

(3) Mapping GPS trajectories: We generate a candidate graph $G_{T}^{\prime }(V_{T}^{\prime },E_{T}^{\prime })$ for each trajectory *TR*: *p*_1_ → *p*_2_ → ⋯ → *p*_*n*_, where $V_{T}^{\prime }$ is the set of candidate points and $E_{T}^{\prime }$ the set of shortest paths between any two neighboring candidate points. The candidate path *P*_*c*_ of GPS trajectory *TR* is denoted $P_{c}=c_{1}^{s_{1}}\to c_{2}^{s_{2}}\to \cdots \to c_{n}^{s_{n}}$, and evaluated by the spatial analysis function $F(P_{c})=\sum_{i=2}^{n}F(c_{i-1}^{s_{i-1}}\to c_{i}^{s_{i}})$. The candidate path with the highest *F* score is the mapping path of trajectory *TR*. The travel time of the path is the time interval between trajectory points *p*_1_ and *p*_n_.

Here, we generate travel paths satisfying the following requirements: a) all trajectory points locate in a specified area with limited space, which ensures appropriate length of each path; b) the sequence of each group of trajectory points is time-ordered and recorded by the same GPS receiver; and c) the time interval between consecutive trajectory points should be less than 45 s (one or two GPS probe readings might be missed by the GPS receivers during recording, so 45 s was set to cover this error).

### Detecting path with anomalous travel time

**Fig 1:**
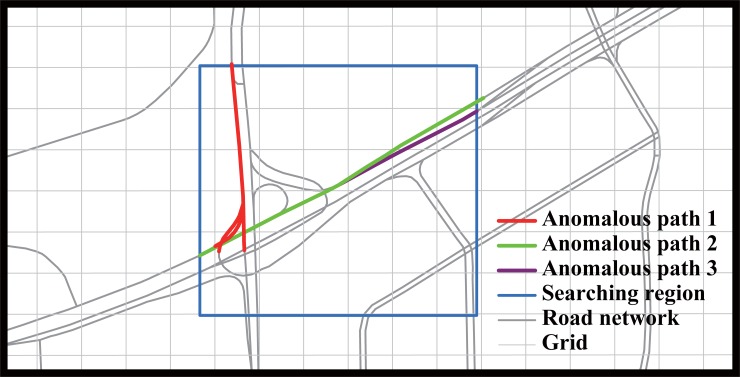
Example of traffic anomaly detection using taxi GPS data. Blue rectangular area confines the region for detecting anomalous paths; colored paths (red, green and purple) illustrate anomalous paths.

The prominent feature of a traffic anomaly is the slowed vehicle speed or the increased travel time. Some practices only used static information, such as road attributes and speed limits to determine whether a road segment is in anomalous traffic state. However, this kind of method fails to discriminate non-recurring anomalies from recurring anomalies. Recurring traffic anomalies are usually caused by the unbalanced relationship between human travel demand and road capacity supply, which cannot be solved in a short time. On the contrary, non-recurring traffic anomalies, caused by unexpected incidents, can be controlled or mitigated by taking proper and timely countermeasures. In this paper, we used historical records to identify non-recurring traffic anomalies and focused only on the analysis of non-recurring traffic anomalies. The reason we choose path travel time for anomaly detection is that road-segment-level traffic anomalies are usually caused by ordinary variations of traffic conditions rather than by accidental traffic events. An example of locating anomalous paths in a searching region is illustrated in Fig 1. Searching regions with anomalous paths detected are defined as anomalous regions. Because some parallel road segments in the studied high-resolution road network belong to the same road in real life, we consider paths that pass through the same sequence of 100 m × 100 m grids as the same path. Therefore, a path within a searching region is defined by a sequence of grids. Obviously, popular routes can have abundant travel time records.

The density based spatial clustering of applications with noise (DBSCAN) algorithm [56] was used to cluster historical travel time records of a path. First, two input parameters, epsilon (EPS) and minimum number of points (MinPts), were determined. The parameter EPS defines the eps-neighborhood scope of a data point and the parameter MinPts is the minimum number of points within the eps-neighborhood scope. According to reference [56], the value of MinPts was set to 4, and the value of EPS was determined as follows. We calculated the EPS value for each path every 30 minutes. The average path-travel-time records of each day in the same 30-min time intervals were grouped and normalized by the largest value during the time interval. Here, only paths with more than 15 historical records at the same time interval were considered, and the distances between any two records of a group were calculated. We used Euclidean distance to calculate the distance between the two normalized path-travel-time records *t*_1_ and *t*_2_:

$$dist(t_{1},t_{2})=|t_{1}-t_{2}|$$ (4)

The fourth-nearest distances of each record to other records were used to fit an exponential probability distribution function. We used the fourth-nearest distance, in which the slope of the exponential function equals to -1, as the value of parameter EPS. For each 30-min time interval, we calculated the average path travel time 〈*t*〉 and the standard deviation *σ* of the largest cluster. Path travel time records exceeding 〈*t*〉 + 3*σ* during the time interval are regarded as anomalous records. Paths with anomalous travel time records are defined as anomalous paths.

## Results

### Data fusion framework for physical and social transportation data

Although traffic anomalies can be identified using taxi GPS trajectory data, it is difficult to infer the reason for such anomalies. Encapsulated with a rich description of traffic events, Weibo messages can help understand the reason for traffic anomalies and decide what proper countermeasures to take. Yet, there are several difficulties in mining traffic events from Weibo data: 1) it is difficult to obtain accurate location by geocoding location entities (i.e., road names and landmarks) of Weibo messages; 2) Weibo messages are usually posted with delay; 3) social media users have different descriptions for the same traffic event in terms of location and influential scale.

**Fig 2:**
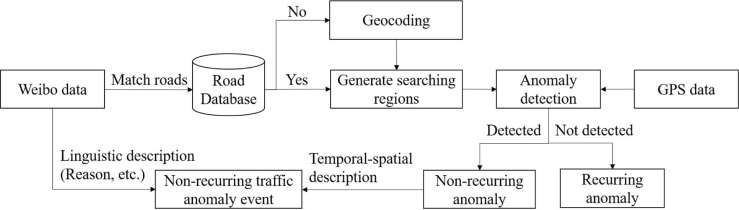
Framework for fusing traffic information from physical and social transportation data.

We propose a data fusion framework to take advantage of both social media data and taxi GPS data (Fig 2). Location entities extracted from Weibo data can be categorized into two types: road names and landmarks. Landmarks are easy to locate because they can be confined to a specific area. Road names, however, usually represent roads with a length of several kilometers. Event location cannot be easily pinpointed using the social media information alone in this case. To deal with this, taxi GPS data are used to identify the anomalous paths along the studied road. First, we retrieve all road segments of the studied road by matching the road name with the database of road information. If no record was matched, we used the location tag or the manually identified location entity as the landmark. Second, multiple searching regions were generated along the target road to detect traffic anomalies. If multiple roads were recognized, we generated searching regions for each target road. For social transportation data with only landmarks recorded, the landmark was transformed into a GPS coordinate using an online geocoding service. A 1000 m × 1000 m rectangular area around the geocoded location was generated as the searching region. Third, if any anomaly is detected, we regarded the traffic event reported by Weibo as a non-recurring event; otherwise, we assumed that the Weibo message referred to a recurring traffic anomaly. Finally, since traffic events reported on social media platforms have been connected with anomalous traffic states, a piece of traffic information with a detailed linguistic description and accurate temporal-spatial description was obtained.

### Case studies

Here, we applied the proposed data fusion framework to three traffic events reported from Weibo messages with road names and one traffic event from Weibo messages without road names. As shown in Table 3, Cases 1–3 include Weibo messages with road names recorded, whereas Case 4 is a Weibo message with only the landmark recorded. Different types of traffic events, from car crashes to disabled cars to road construction, are presented. All Weibo messages reported traffic jams, but the traffic jams are caused by different factors. We can also see that the reasons for traffic jams are often provided in social media messages, and are helpful for deploying effective countermeasure.

**Table 3:** Three types of traffic events reported by Weibo.

Case no.	Post time	Weibo messages (translated into English)	Event type
Case 1	18:41 Oct. 9	[***North Central Avenue***] a faulty car occupied the second lane near Yinhu flyover (eastbound).	Disabled car
Case 2	08:14 Oct. 9	At approximately 8:10, two cars crashed in the middle lane of ***North Wenjin Road*** near Yayuan Overpass (southbound), resulting in a traffic jam.	Car crash
Case 3–1	17:22 Oct. 9	[***Meiguan Road***] one lane of the ramp connecting ***Meiguan Road*** and ***Caitian Road*** is closed due to road construction, slowing traffic.	Road construction
Case 3–2	17:50 Oct. 9	17:45, a car crash on ***Meiguan Road*** at Nanping Bridge (southbound) occupied the rightmost lane, slowing traffic.	Car crash
Case 4	8:59 Oct. 13	A disabled car at ***Caimei overpass*** (southbound) occupied one lane and slowed down the traffic.	Disabled car

**Fig 3:**
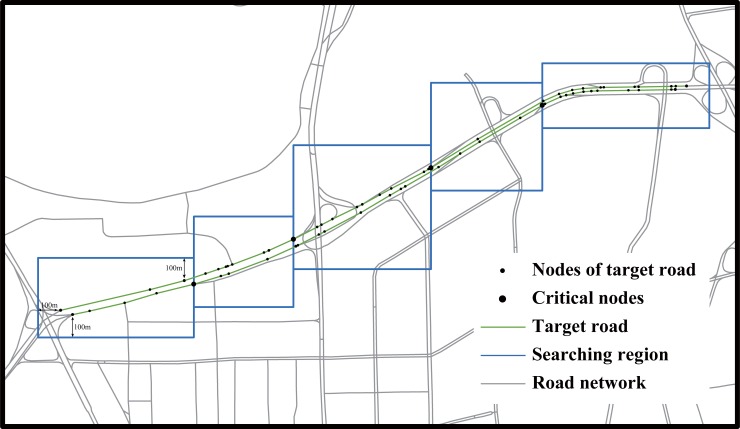
Illustration of method of generating searching regions. Green paths are the target road referred to in the Weibo message; blue rectangles represent generated searching regions.

In social media messages, there are sometimes landmarks recorded to offer more description about the event location with road names, e.g., the landmark “Yinhu flyover” in Case 1 and the landmark “Yayuan Overpass” in Case 2. In previous studies, some researchers used joint words, such as “from…to” and “between…and,” to identify such location information [54,57]. However, this rule-based method lacks flexibility, and the locations may not be the exact locations, but rather the nearest landmarks that social media users can refer to. Road descriptions like “North Wenjin Road” can be recognized by computers, but the exact location and influential scale of the event are unknown due to the fuzziness of human language and the limited length of Weibo posts. Hence, we present an approach to divide a road into several sections and detect the location of anomalous sections in this study:

1. In the studied road network, critical nodes connecting more than two road segments (neighboring critical nodes are confined to be more than 500 m apart) are used to divide the target road into several sections. For each section, the maximum and minimum longitudes and the maximum and minimum latitudes of nodes are used as geographical references to determine the boundaries of the searching regions (Fig 3).
2. To ensure that the searching region covers all potential road segments where a traffic event (reported in social media messages) occurred, we extend the original boundary by 100 m to generate new searching regions.

We analyzed GPS records in six 10-min time windows before and after the time window in which the social media message was posted. The state of each searching region *i* in time window *t* can be described as

$${SR}_{i}(t)=\left\{ \begin{array}{l} 0,\;no\;anomalous\;path\;detected\\ 1,\;anomalous\;path\;detected \end{array}\right.$$ (5)

where *t*ϵ{*T* − 6,…,*T*,…,*T* + 6}, with *T* the post-time window of the Weibo message.

We used two analytic tools, the anomaly index *R* and the anomaly matrix, to capture the temporal-spatial influence caused by a traffic event, as follows.

The anomaly index *R* of a road segment *e* is defined as

$$R_{e}=1-\frac{v_{e}(t)}{{\bar{v}}_{e}(t)}$$ (6)

where *v*_*e*_(*t*) is the average vehicle speed of road segment *e* at time window *t*, and ${\bar{v}}_{e}(t)$ the monthly workday average vehicle speed of road segment *e* at time window *t*. We calculate the values of anomaly indexes *R* for road segments within the searching regions, focusing only on road segments with average vehicle speeds lower than the monthly averages. The anomaly index *R* becomes larger when the vehicle speed *v*_*e*_(*t*) becomes smaller, indicating a worse traffic situation.

The anomaly matrix is defined as

$$A=\left\{sr_{it}\right\},\;sr_{it}=SR_{i}\left(t\right)$$ (7)

where entries of anomaly matrix *sr*_*it*_ are binominal values *SR*_*i*_(*t*). Using an anomaly matrix, we can see how many searching regions are affected in a time window, and how many time windows the anomaly lasts in one searching region. The anomaly period is defined as the beginning of the time when an anomaly is first detected in a searching region to the time window when an anomaly is last detected in a searching region.

**Fig 4:**
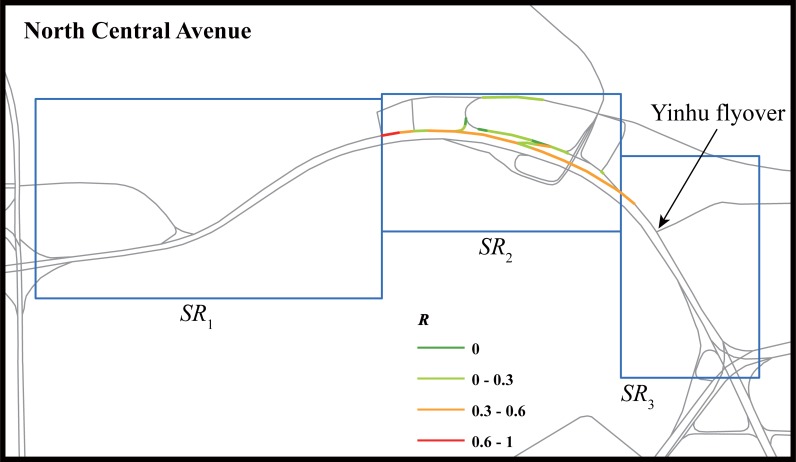
Anomaly index of road segments within searching regions during time window when a social transportation message was posted (Case 1).

Using these methods, we analyzed Case 1–3 shown in Table 3 (Case 3 contains two Weibo messages because they are both about the same road during the same hour). We first present the results of a traffic anomaly caused by a disabled car on North Central Avenue. The anomaly index *R* of road segments during the Weibo post window are shown in Fig 4. The anomaly matrix is shown in Table 4. This traffic incident was first detected in searching region *SR*_3_ at five time windows before the Weibo message was posted. The traffic incident lasted for three time windows in searching region *SR*_3_. After the congestion was alleviated in searching region *SR*_3_, the anomalous traffic state spread to searching region *SR*_2_ when the Weibo message was posted. This traffic event caused a traffic anomaly of a relatively small scale and did not massively influence the traffic conditions in the road network. It was interesting to find that the Weibo message was posted in the last anomalous time window when traffic conditions had begun to rehabilitate. This reveals the property of the delay of Weibo messages.

**Table 4:** Traffic anomaly matrix of Case 1.

*SR*_*i*_(*t*)	T−6	T−5	T−4	T−3	T−2	T−1	T	T+1	T+2	T+3	T+4	T+5	T+6
*SR*_1_	0	0	0	0	0	0	0	0	0	0	0	0	0
*SR*_2_	0	0	0	0	0	**1**	**1**	0	0	0	0	0	0
*SR*_3_	0	**1**	**1**	**1**	0	0	0	0	0	0	0	0	0

**Fig 5:**
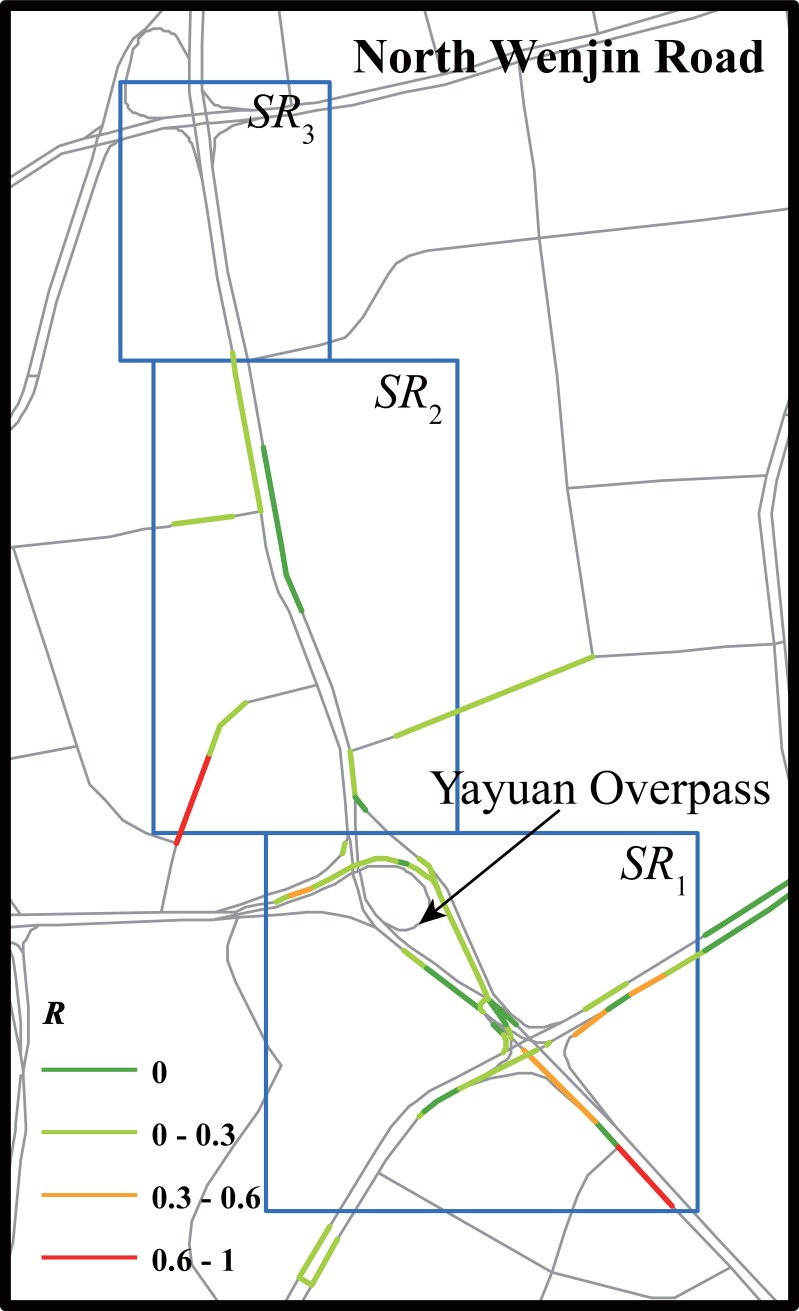
Anomaly index of road segments within searching regions during time window when a social transportation message was posted (Case 2).

The second social transportation message recorded a traffic anomaly caused by a car crash accident on North Wenjin Road. The influence of the car crash accident on traffic was obviously larger than the disabled car in Case 1. A traffic anomaly was first detected four time windows before the Weibo post. The influence of the traffic accident was not eliminated three time windows after the Weibo message was posted (Table 5). More seriously affected road segments were observed during the time window when the Weibo was posted. The affected road segments were not only on the target road, but had also spread to peripheral areas (Fig 5).

**Table 5:** Traffic anomaly matrix of Case 2.

*SR*_*i*_(*t*)	T−6	T−5	T−4	T−3	T−2	T−1	T	T+1	T+2	T+3	T+4	T+5	T+6
*SR*_1_	0	0	**1**	**1**	**1**	**1**	**1**	0	**1**	**1**	0	0	0
*SR*_2_	0	0	**1**	**1**	**1**	**1**	**1**	0	0	0	0	0	0
*SR*_3_	0	0	0	0	**1**	**1**	0	0	0	0	0	0	0

**Fig 6:**
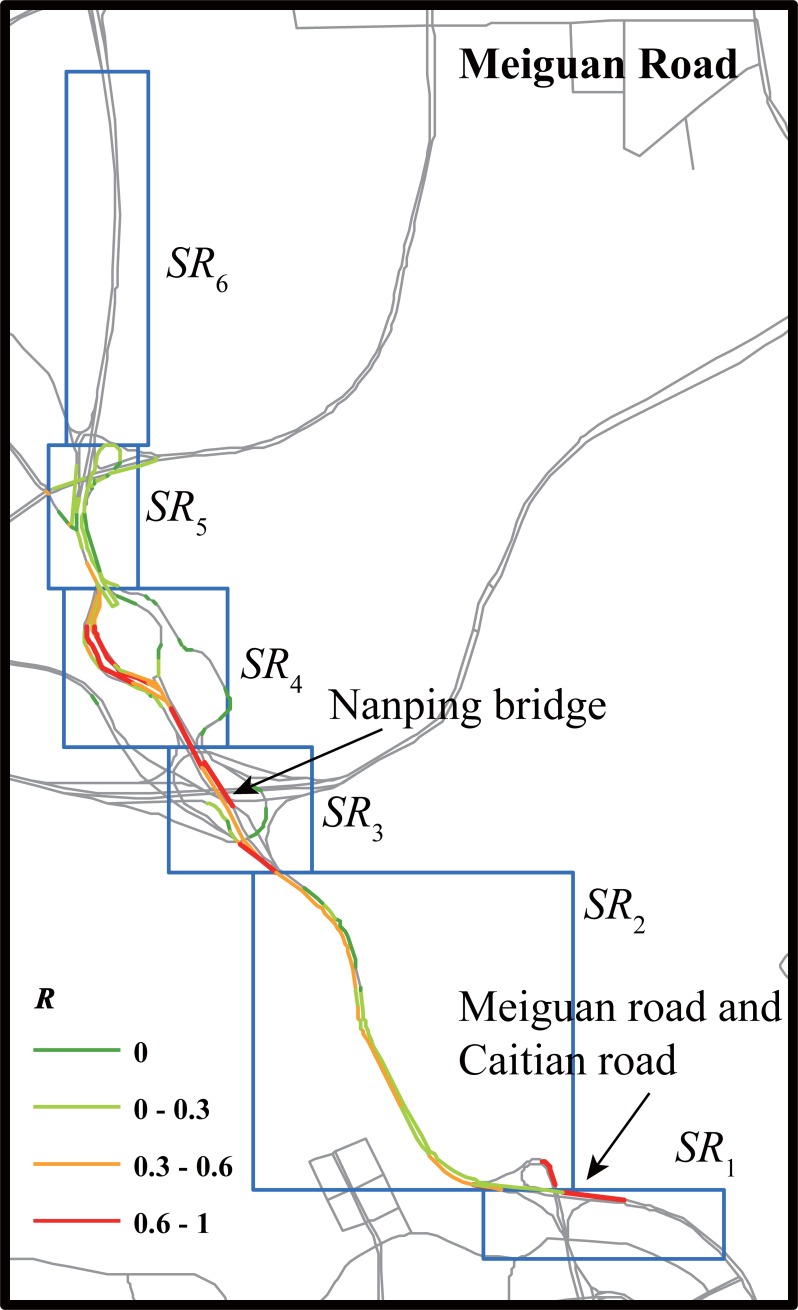
Anomaly index of road segments within searching regions during time window when social transportation messages were posted (Case 3).

We finally present a massive traffic anomaly caused by multiple traffic events on Meiguan road. The traffic anomaly lasted for nearly 2 h and influenced traffic conditions on almost the entire target road (Table 6). There were two Weibo messages describing the traffic anomalies of this road. The first message reported a traffic regulation due to road construction at time window *T*, and the second message reported a car crash accident at time window *T*′(*T* + 2). The first Weibo message was posted in the time window when the traffic state reached the worst situation. During time window *T*, searching regions covering two locations referred in the Weibo messages exhibited the most serious congestion, implying that our method can potentially infer the event location to some degree (Fig 6).

**Table 6:** Traffic anomaly matrix of Case 3.

*SR*_*i*_(*t*)	T−6	T−5	T−4	T−3	T−2	T−1	T	T+1	T+2	T+3	T+4	T+5	T+6
*SR*_1_	0	0	0	0	0	0	0	0	0	0	0	0	0
*SR*_2_	0	**1**	**1**	**1**	**1**	**1**	**1**	**1**	0	0	0	0	0
*SR*_3_	**1**	**1**	**1**	**1**	**1**	**1**	**1**	**1**	**1**	**1**	**1**	**1**	0
*SR*_4_	0	0	0	**1**	**1**	**1**	**1**	**1**	**1**	0	0	0	0
*SR*_5_	**1**	**1**	**1**	**1**	0	0	**1**	**1**	**1**	0	0	0	0
*SR*_6_	0	0	0	0	0	0	0	0	0	0	0	0	0

**Fig 7:**
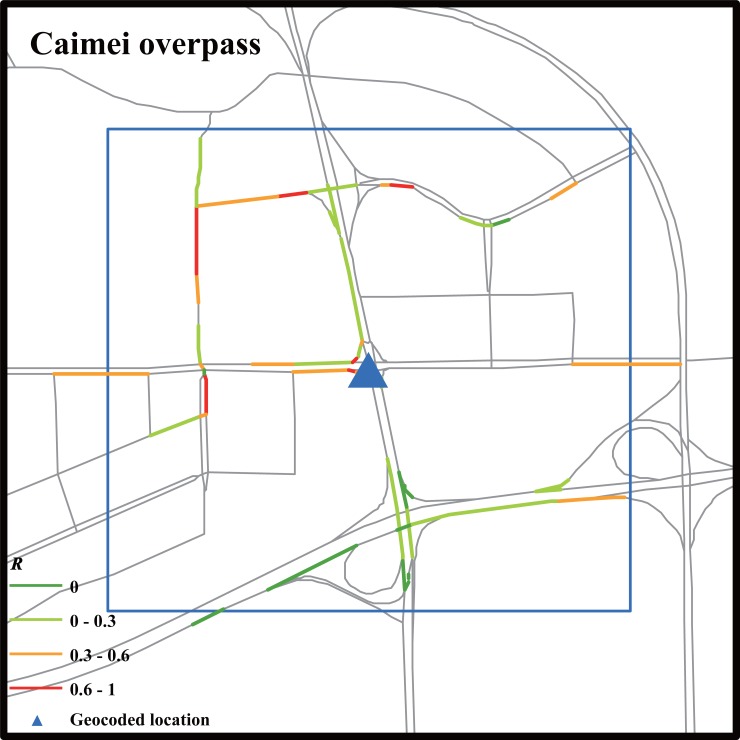
Anomaly index of road segments within searching regions during time window when a social transportation message was posted (Case 4). The blue triangle is the geocoded location of the landmark referred in the Weibo message.

For Weibo messages without recording road names (Case 4), we searched anomalous paths in a 1000 m × 1000 m rectangular area. Weibo users do not report a road name of a traffic event probably because the traffic event occurred exactly at a landmark or closely nearby. Such a situation occurs less frequently than traffic events occurring at locations without landmarks nearby, which can be seen in the comparison of the numbers of collected Weibo messages (99 versus 502). As shown in Table 7, the traffic anomaly completely dissipated when the Weibo message was posted, showing that Weibo data sometimes lack time efficiency. Comparatively, the traffic state inferred from GPS data showed an anomaly four time windows ahead of the Weibo data and offered the spatial distribution of affected road segments (Fig 7).

**Table 7:** Traffic anomaly matrix of Case 4.

*SR*_*i*_(*t*)	T−6	T−5	T−4	T−3	T−2	T−1	T	T+1	T+2	T+3	T+4	T+5	T+6
*SR*_1_	0	0	0	**1**	**1**	**1**	0	0	0	0	0	0	0

### Statistical analysis

As illustrated in the Case studies, we employed similar methods to analyze the identified 601 Weibo messages. There were 502 Weibo messages containing road names and 99 Weibo messages with landmarks only, implying that road names are the easiest location entity people can refer to. We detected non-recurring traffic anomalies for 350 of the 502 Weibo messages with road names. Only 64 out of the 99 Weibo messages without road names could be successfully geocoded. The geocoding failures could be caused by the ambiguous descriptions of landmarks. The online map service may not be able to identify the location of corresponding Point of Interest (POI). This finding also implies that Weibo users sometimes cannot describe the location appropriately. Non-recurring traffic anomalies were detected in 35 of the 64 geocoded messages.

**Fig 8:**
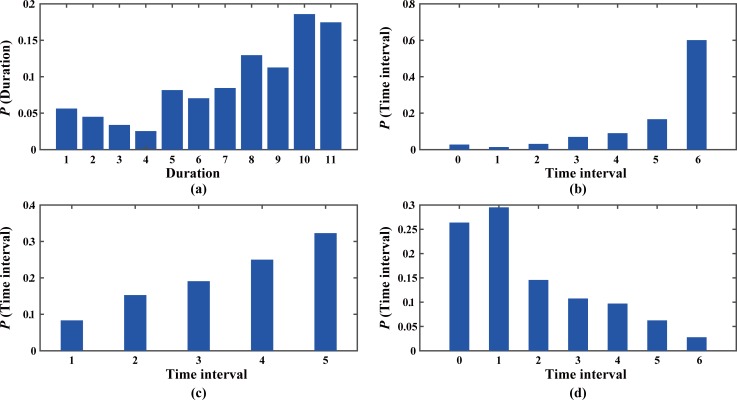
Temporal analysis of traffic events. (a) The probability distribution of duration of anomalies. The probability distribution of time interval between Weibo post window and (b) the beginning of the anomaly period, (c) the end of the anomaly period and (d) the time window with most anomalous regions among Weibo messages posted during a traffic anomaly.

We focused on the efficiency of 350 detected anomalies reported by Weibo messages with road names. The distribution of duration of anomalies is shown in Fig 8(A). More than half of non-recurring traffic anomalies last for more than 1 h, implying that non-recurring traffic events usually lead to severer traffic disruption. We then separated Weibo messages into three types: 1) Weibo messages posted before a traffic anomaly; 2) Weibo messages posted during a traffic anomaly; 3) Weibo messages posted after a traffic anomaly. Results show that only 11 of the 350 Weibo messages were posted before a traffic anomaly, while 288 of the 350 Weibo messages were posted during a traffic anomaly. Additional efficiency analysis of GPS data and Weibo data were carried out among 288 Cases. Most Weibo messages were posted 60 mins after the beginning of the anomaly period [Fig 8(B)], while, from another point of view, they are not too late because most are still 30 mins ahead of the end of the anomaly period [Fig 8(C)]. More importantly, we calculated the time interval between the Weibo post window and the time window with most anomalous regions [Fig 8(D)]. Most Weibo messages are posted in or closely around the time window with the largest traffic disruption. The results show that GPS data out-perform Weibo data in terms of time efficiency, while Weibo data can provide supplementary information about the significant traffic disorder during the most urgent situation.

## Discussion and conclusions

In this study, we present a data fusion framework for detecting high-priority traffic information from social transportation data. This framework incorporates several analytical tools to capture multi-dimensional traffic anomaly information from taxi GPS data and social media data. Case studies of different types of traffic anomalies are analyzed. The results prove the effectiveness of our framework in evaluating the influential scale and in validating the reality of non-recurring traffic anomalies reported by Weibo messages. Statistical analysis shows the efficiency of our framework in capturing traffic anomalies of different scales. Weibo data then provide valuable descriptions of traffic events to illustrate anomalies. Our framework leverages the advantage of both physical transportation data and social transportation data and inspires a bright future for similar research.

However, future research is required to further understand the features of different traffic anomalies. First, the generation and dissipation of traffic anomalies are complicated processes that vary with different triggering events. Hence, the pattern of anomalies should be analyzed temporally and spatially to more accurately locate the incident. Second, more tools are needed to discriminate traffic anomalies when multiple incidents occur on the same road. Finally, the framework illustrated in this paper can be easily extended to multiple data sources, and can provide richer and more accurate information about traffic anomalies.

This study mainly investigates the potential of social media data in traffic and transportation analysis. In future works, more data mining techniques can be incorporated into the present framework to collect traffic and transportation information in an automatic manner. For example, the transport-related Weibo messages are manually identified in this study. However, this process can be automatically conducted using a number of classification tools (e.g. support vector machine (SVM) [1]). In addition, named entity recognition (NER) [2] can be used to identify location entities when no road names nor location tags are provided.

One difficulty for applying the presented framework in an automatic manner is the lack of methods that can automatically extract the reasons of traffic events and the suggestions of travelers from social media messages. To our best knowledge, there is no dedicated method to accomplish this goal. Although the present study uses human resource to parse the textual information, it still represents a feasible approach in practices. During the 19 days of data collection in Shenzhen, totally 385 non-recurring traffic anomalies are identified. Parsing roughly 20 Weibo reports per day will not generate too much extra work for traffic regulators, and the obtained information is useful for understanding the reason of accidental traffic event and take suitable countermeasures.

## Supporting information

S1 Dataset The minimal dataset to replicate this study. (ZIP)
